# Biotechnological Potential of *Cephalaria uralensis* (Murray) Roem. & Schult. and *C. gigantea* (Ledeb.) Bobrov—Comparative Analysis of Plant Anatomy and the Content of Biologically Active Substances

**DOI:** 10.3390/plants10050986

**Published:** 2021-05-15

**Authors:** Małgorzata Chrząszcz, Katarzyna Szewczyk, Dorota Tchórzewska

**Affiliations:** 1 Department of Pharmaceutical Botany, Medical University of Lublin, 1 Chodźki Str., 20-093 Lublin, Poland; malgorzata.chrzaszcz329@gmail.com; 2 Department of Cell Biology, Institute of Biological Sciences, Maria Curie-Skłodowska University, Akademicka 19, 20-033 Lublin, Poland

**Keywords:** Caprifoliaceae, *Cephalaria*, morphology, histology, primary metabolites, phenolic, flavonoids, phenolic acids

## Abstract

Studies conducted to date have shown that *Cephalaria uralensis* and *C. gigantea* have high contents of substances with antibacterial, anti-inflammatory, and antioxidant properties; hence, they are attractive plants from the pharmaceutical point of view. However, despite their multifarious desirable biotechnological aspects, the knowledge of these plants is insufficient. The present study focused on the analysis of the morphological, anatomical, and histological structure of aboveground parts of the plants, the identification of the distribution of biologically active compounds in the tissues, and quantitative phytochemical analyses of polyphenolic compounds contained in their aboveground organs. Importantly, the phenological and morphological features of the aboveground organs in the analyzed species were maintained, as in the same plant species growing in different climatic conditions. The analysis of primary metabolites and phenolic compounds in the tissues revealed their distribution in the aboveground organs, which has never been described before. The comparative analyses of the content of total phenolics, total phenolic acids, and total flavonoids in the aboveground organs showed that the level of these substances differed not only between the species but also between the organs. It should be emphasized that the level of these compounds is higher than in many other medicinal plants.

## 1. Introduction

The family Caprifoliaceae comprises herbaceous plants (annuals, biennials, and perennials), climbers, shrubs, and trees. This relatively small group of plants (approx. 960 species) has a worldwide distribution range [1], but only a few species are used as medicinal plants. These include, e.g., *Valeriana officinalis*, which is traditionally used for the treatment of insomnia, mild nervous conditions, and menopausal symptoms and is recommended for the treatment of gastrointestinal pain and spastic colitis [2,3], *Lonicera caprifolium* with its diuretic, diaphoretic, and antibacterial effects, and *L. japonica*, used for the treatment of prostatitis and hypertension [2]. It should be emphasized that many Caprifoliaceae species have not yet been analyzed for the presence of high contents of secondary metabolites and their broad-sense medicinal use.

One of the poorly known groups of plants from the family Caprifoliaceae is the genus *Cephalaria*, whose species usually grow on dry xerothermic slopes and steppes [4]. It comprises approximately 100 species, including 24 endemic species occurring only in Turkey. This genus is especially widespread in the Balkan Peninsula, the Mediterranean Region, South Africa, and the Middle East [5,6,7,8]. Species of the genus *Cephalaria* are usually perennial plants with lanceolate or pinnate leaves and spherical inflorescences developing on long, erect shoots [9]. Previous studies have shown that species belonging to the genus *Cephalaria* are rich in iridoids [10,11], flavonoids [10,12], caffeic acid derivatives [10], triterpene saponins [13,14,15,16], and alkaloids [17]. Due to the presence of a variety of chemical constituents, these plants are attractive species from a medicinal perspective. Investigated *Cephalaria* species have been shown to exhibit broad cytotoxic activity [16], e.g., antifungal [18], antiprotozoal [19], and antibacterial [20] effects. They are also rich in many secondary metabolites with antioxidant properties [21].

Given the current challenges to modern medicine, it is extremely important to search for new plant species containing substances that can not only support/supplement current therapies, but also open new therapeutic pathways based on innovative natural medicinal products. This is particularly important given the phenomenon of multi-drug resistance (MDR), which is currently responsible for the huge threat from pathogenic microorganisms rapidly acquiring resistance to antibacterial drugs [22,23,24]. Hence, investigations searching for new antibacterial substances of natural plant origin may contribute to overcoming the phenomenon of the drug resistance of many pathogenic bacteria and increase the effectiveness of medical therapies [25].

One of the most common skin diseases affecting the human population is seborrheic skin diseases, especially acne vulgaris. This disease causes chronic skin lesions mainly in the face area, which may result in the emergence of mental disorders, e.g., depression. This troublesome dermatosis is caused by bacterial infections, which are treated primarily with antibiotics. In addition to the issue of antibiotic resistance and the risk of adverse side effects produced in the organism, antibiotic therapy is associated with the problem of environmental pollution with synthetic drugs and disturbances in the ecosystem. To date, little research has been done to search for plant-origin substances with antibacterial activity in such an extremely difficult-to-treat disease as acne. In our previous study, we screened the biological activities of compounds from *Cephalaria uralensis* and *C. gigantea* and showed that both species contained biologically active antibacterial substances with additional anti-inflammatory and antioxidant properties, but no cytotoxic effects towards human skin fibroblasts were observed [10]. Thus, we have shown that the biologically active compounds contained in *C. uralensis* and *C. gigantea* can reduce inflammation via the inhibition of the growth of pathogenic acne-causing bacteria.

The present study represents a continuation of research on the biotechnological aspects of *C. uralensis* and *C. gigantea* plants and fits in the trend of the search for new species with medicinal applications. To provide a comprehensive view, we focused on several issues, namely, the morphological and histological structure of aboveground shoots in *C. uralensis* and *C. gigantea* and cytochemical analyses for the identification and characterization of the distribution of biologically active compounds in the tissues, which have never been described before in these species. We also performed quantitative phytochemical analyses of polyphenolic compounds contained in the aboveground organs of both species. This wide-ranging study of *Cephalaria* plants provides insight into their structure and the distribution of biologically active compounds in the elements of these plants. The detailed analysis of the aboveground organs of these species indicated the potential of an effective use of the individual parts of these plants for the extraction of biologically active compounds.

## 2. Results and Discussion

### 2.1. Anatomical, Histological, and Cytological Analysis of Cephalaria uralensis

**Figure 1:**
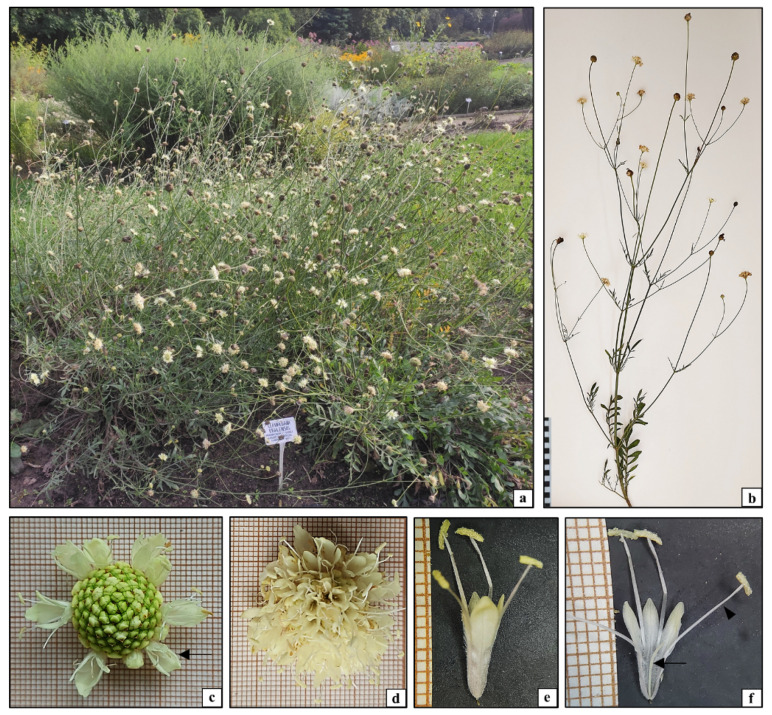
Morphology of *Cephalaria uralensis*: (**a**) cultivation in the botanical garden; (**b**) aboveground parts of plants; (**c**) inflorescence with some flowers in the anthesis stage (arrow); (**d**) inflorescence with all flowers in the anthesis stage; (**e**) single flower in the anthesis stage; (**f**) generative elements of a flower in the anthesis stage: stamen (arrowhead), pistil (arrow).

*Cephalaria uralensis* analyzed in this study is a perennial plant species growing in compact clumps [4]. In the temperate climate, a rosette of lanceolate and pinnate leaves as well as 100 cm to 120 cm high inflorescence stems started growing from underground rhizomes at the beginning of the vegetation season (Figure 1a). The erect stems were characterized by opposite dichotomous branching and leaf arrangement. Each aboveground shoot had a spherical capitulate inflorescence at the apex (Figure 1b). The inflorescences in *C. uralensis* had numerous sessile simple flowers. The flowers in the inflorescence were very densely arranged and exhibited non-synchronous development. They bloomed from July to September. The slightly larger marginal flowers opened first; later, anthesis in the other smaller flowers gradually proceeded towards the center of the inflorescence (Figure 1c,d). The simple flowers had a perianth composed of a serrated calyx (Figure 1c arrow) and a tubular corolla composed of four yellow petals covered by numerous trichomes (Figure 1e). The generative elements in the simple flower included four stamens and an inferior ovary. Mature anthers opened along their entire length. The filaments, anthers, and mature pollen grains were yellow (Figure 1e,f). The phenology and morphological features of the aboveground organs (leaves, stems, and flowers) observed in the analyzed species did not show any major differences from *C. uralensis* plants growing in different climatic conditions, indicating that the features of this plant are not strictly dependent on climate conditions [4,26,27].

**Figure 2:**
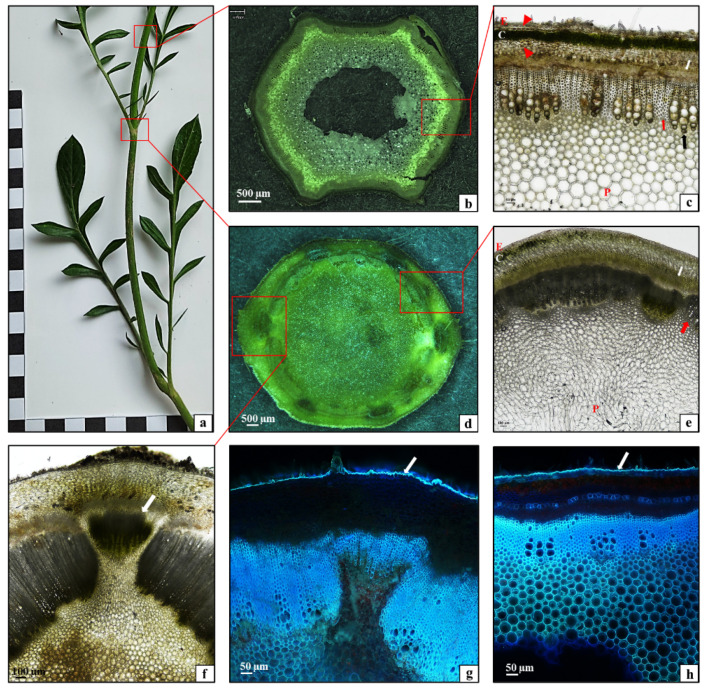
Morphology and anatomy of a *Cephalaria uralensis* stalk: (**a**) fragment of a one-year-old stalk; (**b**,**c**) cross-section through the stalk at the internode part: (**c**) epidermis (E), lamellar collenchyma (arrowhead), assimilation parenchyma (C), phloem (white arrow), xylem (black arrow), sclerenchyma fibers (red arrow), parenchyma (P); (**d**–**f**) cross section of the stalk in the node part: (**e**) epidermis (E), assimilation parenchyma (C), phloem (white arrow), xylem (red arrow), parenchyma core (P); (**f**) arrow—branching vascular bundle reaching the leaf; (**g**,**h**) cuticle layer (arrow) covering the epidermis stalk: (**g**) part of the node, (**h**) part of the internode ((**b**,**d**) stereoscopic microscope; (**c**,**e**) light microscope; (**g**,**h**) fluorescence microscope).

The stem in the *C. uralensis* plants was composed of nodes bearing leaves (Figure 2a,b) and sections between the nodes called internodes (Figure 2a,c). As shown by the analysis of the histological structure of the *C. uralensis* stem internode, the entire circumference of this organ was covered by a single-layered epidermis (Figure 2c—E); underneath, there was a layer of lamellar collenchyma (Figure 2c—arrowhead) and assimilation parenchyma with numerous chloroplasts (Figure 2c—C). Beneath the parenchyma, there was another layer of strengthening tissue—the collenchyma (Figure 2c—arrowhead), with a width directly proportional to the age of the stem. Conducting elements, i.e., the phloem (Figure 2c—white arrow) and the xylem (Figure 2c—black arrow), formed a layer around the stem circumference. Numerous layers of strengthening sclerenchyma fibers were visible in the xylem (Figure 2c—red arrow). The subsequent layer was composed of parenchyma cells (Figure 2c—P). There was a relatively small air canal in the center of the stem (Figure 2c). The microscopic analysis of the stem showed that the entire circumference of the stem nodes was covered by a single-layered epidermis (Figure 2e—E). The subsequent layers were the assimilation parenchyma (Figure 2e—C), the phloem (Figure 2e—white arrow), and the xylem (Figure 2e—red arrow). The core was filled with parenchyma tissue (Figure 2e—P). A branching vascular bundle was visible at the site of growth of the leaf sheath (Figure 2f—arrow). As shown by the fluorescence microscopy analysis of the cuticle covering the epidermis, it formed a small layer with the same thickness on the entire *C. uralensis* stem (Figure 2g,h—white arrows). Therefore, it can be concluded that the histological structure of the stem in the studied species represents a typical tissue system characteristic for this type of organ in dicotyledonous plants [28]. Moreover, it was observed that, although its thickness was directly proportional to the age of the stem, the strengthening tissue layer was relatively thin, which was largely related to the medium length of the aboveground shoots.

**Figure 3:**
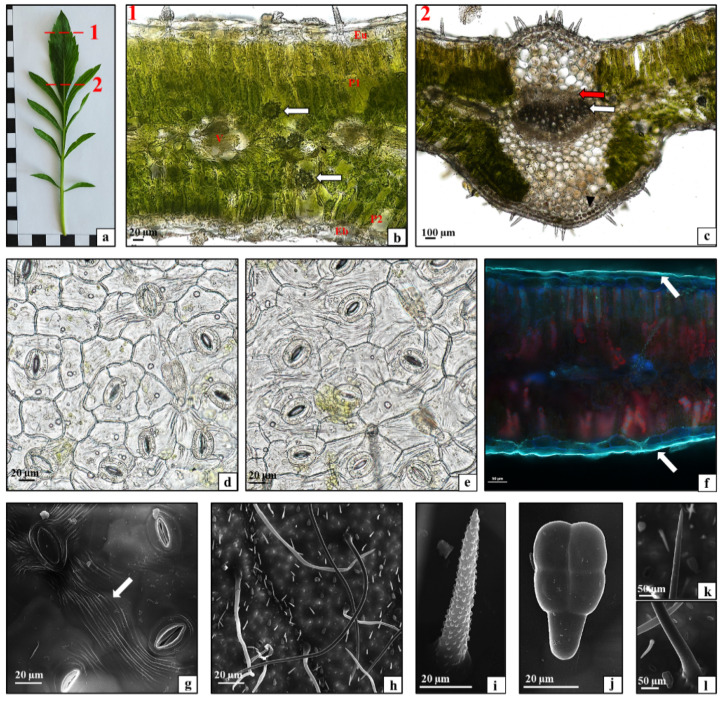
Morphology and anatomy of a *Cephalaria uralensis* leaf: (**a**) macroscopic view, dotted line (1 and 2)—histologically analyzed fragments; (**b**) cross-section through the leaf blade: upper epidermis (Eu), palisade mesophyll (P1 and P2), spongy mesophyll (S), vascular bundle (V), calcium oxalate crystal (arrows), bottom epidermis (Eb); (**c**) cross-section through the midrib: lamellar collenchyma (arrowhead), phloem (red arrow), xylem (white arrow); (**d**) adaxial leaf epidermis; (**e**) abaxial leaf epidermis; (**f**,**g**) cuticle layer on the upper and the lower epidermis (white arrows); (**h**–**l**) trichomes on the lower and the upper side of the leaf blade. ((**b**–**e**) light microscope; (**f**,**g**) fluorescence microscope; (**h**–**l**) SEM).

The *C. uralensis* leaves were pinnate with lanceolate entire or serrated sections (Figure 3a). The cross sections of the leaf blade showed the adaxial (Figure 3b—Eu) and abaxial (Figure 3b—Eb) epidermis, the palisade parenchyma composed of tightly adherent elongated cells arranged in two rows (Figure 3b—P1), and the spongy parenchyma composed of loosely arranged cells with large intercellular spaces (Figure 3b—S). Vascular bundles were visible in the spongy parenchyma (Figure 3b—V). Single-layered palisade parenchyma was located at the abaxial epidermis (Figure 3b—P2). Moreover, the lamina exhibited the presence of numerous calcium oxalate crystals, mainly in the spongy parenchyma (Figure 3b—arrows). The analysis of the anatomical structure of the midrib showed the strengthening tissue, i.e., lamellar collenchyma, under the epidermis layer (Figure 3c—arrowhead) and a large vascular bundle in the center (Figure 3c—red and white arrow). The peel preparations of the epidermis from the abaxial and adaxial surfaces of the lamina showed that the tissue was composed of cells with a more or less parentimatic polyhedral shape. There were numerous diacytic Amaryllis-type stomata (Figure 3d,e). The fluorescence microscopy visualization showed that the cuticle covering the epidermis on the upper and lower sides of the lamina was relatively thin and homogeneous on both surfaces (Figure 3f—white arrows). In turn, characteristic linear ornamentation of the cuticle on both the adaxial and abaxial lamina epidermis was revealed by scanning electron microscopy (SEM) (Figure 3g—white arrow). Three types of trichomes were present on the adaxial and abaxial surfaces of the *C. uralensis* leaf blade (Figure 3h). Some of them were relatively short sharp-pointed mechanical hairs with extensive ornamentation (Figure 3i). There were also multicellular Labiatae-type glandular trichomes consisting of a stalk and a head (Figure 3j) and very long, sharp-pointed protective trichomes (Figure 3k,l). Therefore, with their tissue structure, the leaves of *C. uralensis* represented the isolateral type, as the palisade parenchyma was located on both the adaxial and abaxial sides of the lamina. The leaf structure with the palisade parenchyma covering most of the mesophyll is characteristic of species growing in highly sunlit habitats, as palisade parenchyma cells protect the chlorophyll of the spongy parenchyma cells against photodamage [29,30]. Similarly, the strong ornamentation of the cuticle covering the lamina epidermis, which protects this organ against excessive sunlight and transpiration, proves the anatomical adaptation of the leaves to the arid steppe environment, which is the natural occurrence range of the species [31]. Moreover, the spongy parenchyma contained a large number of calcium oxalate crystals, which together with the numerous trichomes and secretory cells protect the plant against herbivores [32,33].

**Figure 4:**
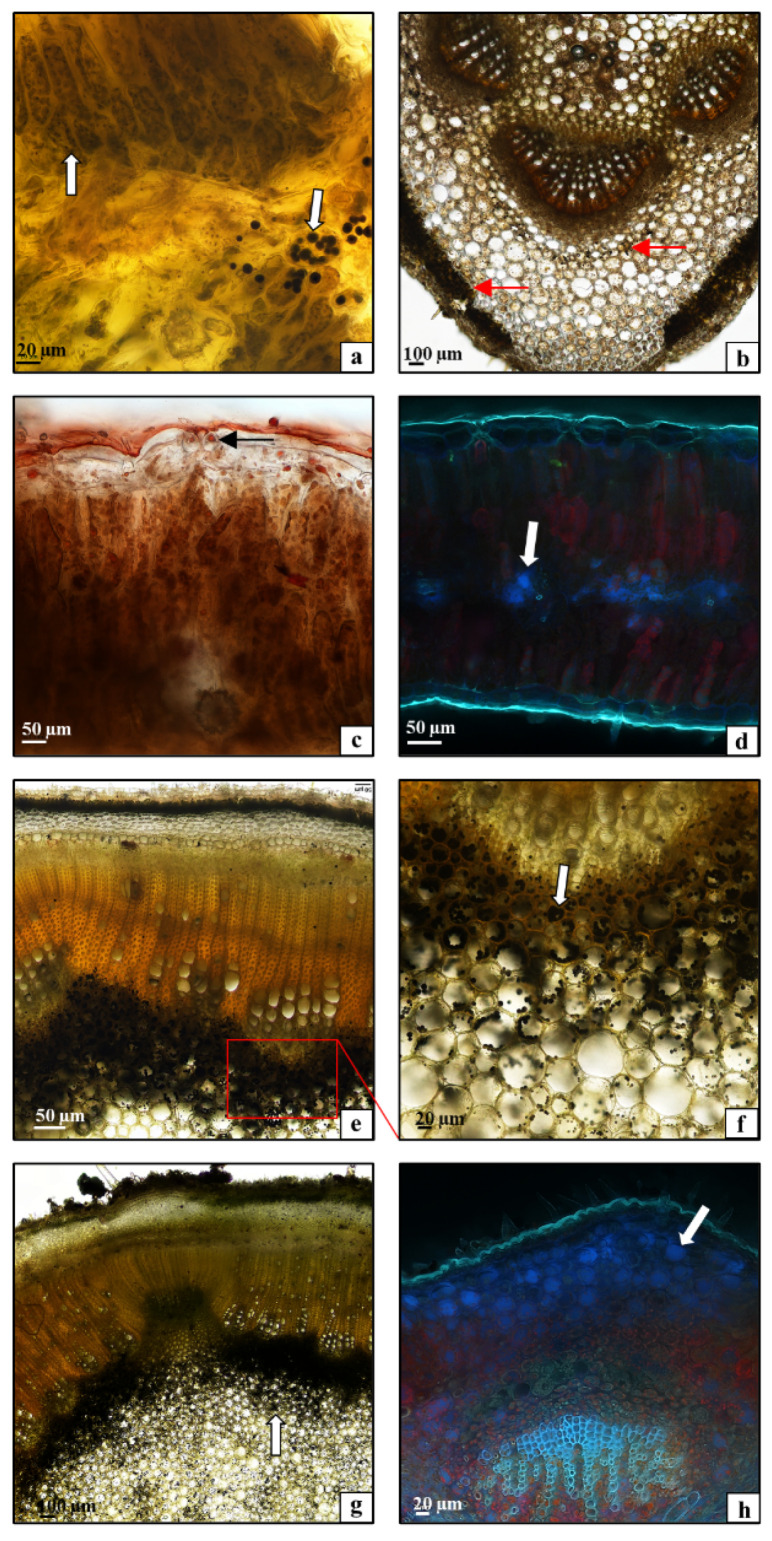
(**a**–**d**) Cross-section through a *Cephalaria uralensis* leaf: (**a**) starch grains (arrows) in the leaf blade lamina; (**b**) starch grains (arrows) in the midrib; (**c**) fat droplets (arrows) in the leaf blade; (**d**) phenolic compounds (arrows) in the leaf blade mesophyll. (**e**–**h**) Cross-section through a *Cephalaria uralensis* stalk: (**e**,**f**) starch grains (arrows) in the internode; (**g**) starch grains (arrows) in the node; (**h**) phenolic compounds (arrows) in the node ((**a**–**c**,**e**–**g**) light microscope; (**d**,**h**) fluorescence microscope).

To visualize starch, sudanophilic fats, and phenolic compounds present in the tissues of *C. uralensis* stems and leaves, cytochemical tests were performed using light and fluorescence microscopes. The analyses demonstrated that starch was accumulated in the spongy parenchyma and chloroplasts of the palisade parenchyma in the leaf blade (Figure 4a—arrows). In contrast, in the midrib area, greater amounts of starch were detected in the mesophyll immediately beneath the epidermis and around the vascular bundle (Figure 4b—arrows). Small numbers of oleosomes containing sudanophilic lipids were observed in the leaf tissues as well (Figure 4c—arrows). The analysis of the distribution of phenolic compounds in the *C. uralensis* leaf tissues showed that they were accumulated in large amounts, mainly in the spongy parenchyma of the lamina (Figure 4d—arrows). In turn, the cytochemical studies of the stem demonstrated accumulation of high amounts of starch in the parenchyma of both the internodes (Figure 4e,f—arrow) and the nodes (Figure 4g—arrow). Similarly, the analyses of phenolic compounds in the stem tissues showed large amounts of these substances in the parenchyma immediately beneath the epidermis (Figure 4h—arrow). To sum up, it can be concluded that the distribution of the chemical compounds detected in this study with the use of cytochemical methods (starch, lipids) and analyzed based on physicochemical properties (phenolic compounds) is correlated with the characteristic structure of the leaves and stems. Namely, the isolateral *C. uralensis* leaves accumulated starch in spongy parenchyma, palisade parenchyma plastids, and the midrib. Large amounts of starch were also detected in the parenchyma core in the stem internodes and nodes. The relatively high content of starch in *C. uralensis* tissues is important because it is involved in the mechanism of regulation of carbon reserves necessary for plant metabolism and growth [34]. Thus, it can be concluded that the spongy parenchyma is the main reservoir of phenolic compounds in leaves. In the stem, large amounts of these compounds were located in the cortex parenchyma. The present analyses provide the first insight into the distribution of phenolic compounds in the tissues of aboveground organs in species from the family Caprifoliaceae.

### 2.2. Anatomical, Histological, and Cytological Analysis of Cephalaria gigantea

**Figure 5:**
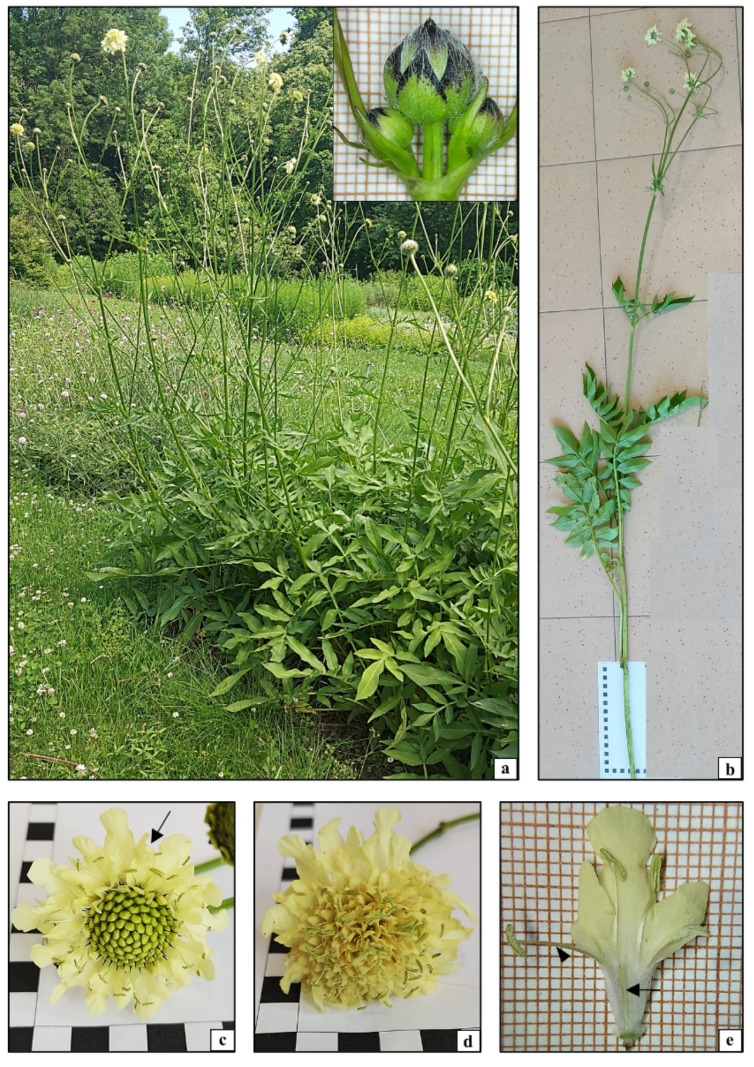
Morphology of *Cephalaria gigantea*: (**a**) cultivation in the botanical garden, young flower buds (small picture); (**b**) aboveground parts of plants; (**c**) inflorescence with some flowers in the anthesis stage (arrow); (**d**) inflorescence with all flowers in the anthesis stage; (**e**) generative elements of a single flower in the anthesis stage: stamen (arrowhead), pistil (arrow).

The other species analyzed in the present study was *Cephalaria gigantea*, i.e., a perennial species from the family Caprifoliaceae. In spring, the underground rhizomes of this species produce rosettes of pinnate leaves and inflorescence stalks reaching a height of 150 cm to 250 cm (Figure 5a). Both the leaves and lateral shoots were arranged dichotomously on erect stems. The apex of each shoot had a spherical capitulate inflorescence covered by densely pubescent overlapping involucre bracts (Figure 5a—small picture; Figure 5b). The species flowered between June and August. The *C. gigantea* inflorescences had numerous, densely arranged, simple flowers characterized by non-synchronous development. The anthesis was first reached by marginal flowers, which were substantially larger than the flowers in the center of the inflorescence opening later (Figure 5c,d). The simple flower had a tubular yellow corolla composed of four petals with abundant trichomes. Four stamens and an inferior ovary were the generative elements of the flower. All elements were yellow and mature anthers opened along their entire length (Figure 5e). The phenology of *C. gigantea* and the morphological features of its leaves, stems, and flowers indicated no large differences from plants of the species growing in different climatic conditions [4,26,27], once again showing that the development of this species is not fully dependent on climate conditions. Thus, this information indicates that this plant can be cultivated in a wide range of conditions, which emphasizes its applicability in agriculture and crop production for pharmaceutical needs.

**Figure 6:**
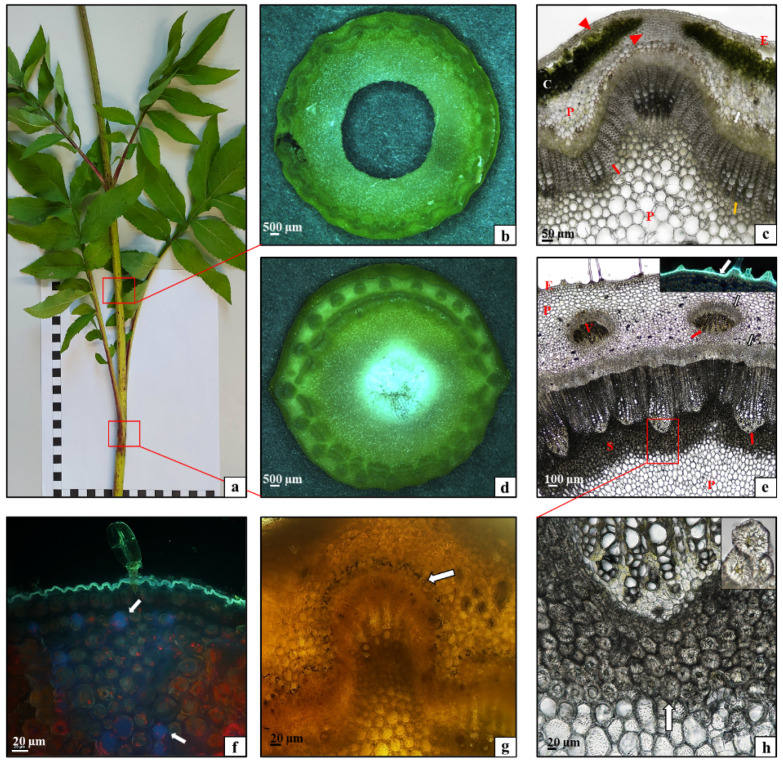
Morphology and anatomy of a *Cephalaria gigantea* stalk: (**a**) fragment of a one-year-old stalk; (**b**,**c**) cross-section through the stalk at the internode part: (**c**) epidermis (E), collenchyma (arrowheads), assimilation parenchyma (C), parenchyma (P), phloem (white arrow), xylem (yellow arrow); (**d**,**e**) cross section of the stalk in the node part: (**e**) epidermis (E), parenchyma (P), vascular bundle (V), phloem (white arrows), xylem (red arrows), sclerenchyma (S), small picture—cuticle layer; (**f**) phenolic compounds (arrows) in the internode; (**g**) starch grains (arrows) in the internode; (**h**) fragment of sclerenchyma from the node—stone cells (arrow and small picture). ((**b**,**d**) stereoscopic microscope; (**c**,**e**,**g**,**h**) light microscope; (**e**) small picture, (**f**) fluorescence microscope).

The analysis of the anatomical structure of the *C. gigantea* stem showed that this organ was composed of nodes (Figure 6a,b) and internodes (Figure 6a,d). The internodes were covered by a single-layered epidermis along its entire circumference (Figure 6c—E); underneath, there was a layer of lamellar collenchyma, and a thicker layer of such collenchyma was present above large conducting elements (Figure 6c—arrowheads). Assimilation parenchyma with numerous chloroplasts was visible under the strengthening tissue layer (Figure 6c—C), and parenchyma cells were visible underneath (Figure 6c—P). Conducting elements, i.e., the phloem (Figure 6c—white arrow) and the xylem (Figure 6c—yellow arrow), formed a layer around the stem circumference. Numerous layers of strengthening sclerenchyma fibers were visible in the xylem in addition to vessels (Figure 6c—red arrow). Parenchyma cells formed the subsequent layer (Figure 6c—P) and there was a relatively small air canal in the center of the stem (Figure 6c). The stem in the area of the nodes had a slightly different structure. A thick layer of parenchyma cells was visible under the single-layered epidermis (Figure 6e—E) covered by a relatively thick cuticle (Figure 6e—small picture, white arrow). The parenchyma contained single vascular bundles arranged along the entire circumference of the stem (Figure 6e—V). The phloem (Figure 6e—white arrows) and the xylem (Figure 6e—red arrows) formed a ring of vascular tissue, to which a wide layer of sclerenchyma stone cells adhered on the side of the parenchyma core (Figure 6e—red square; Figure 6h—white arrow and small picture). It can therefore be concluded that the anatomical structure of the *C. gigantea* stem was typical for stems of dicotyledonous plants [28]. However, this organ was characterized by a relatively large layer of strengthening tissue in the xylem in the area of the internodes and a very wide layer of this tissue in the node area. Such a large amount of sclerenchyma in the stem is necessary for supporting the erect aboveground shoots of the species, which can reach a considerable height (even up to 250 cm). The observation of the autofluorescence of phenolic compounds in the stem of the studied species indicated the accumulation of large amounts of these substances in the sub-epidermal parenchyma in the zones between the assimilation parenchyma (Figure 6f—arrows). Small amounts of starch were present in the parenchyma along the entire circumference of the stem internode immediately above the conducting elements (Figure 6g—arrow). Notably, the content of starch in the stem parenchyma tissue in *C. gigantea* was substantially lower than that in the same organs of *C. uralensis* examined in this study. Additionally, as shown by the analysis of the distribution of phenolic compounds in the tissues of the *C. gigantea* stem, the storage parenchyma accumulating these compounds occupied a smaller area than in *C. uralensis*. This is probably also associated with the fact that a large part of the stem diameter in *C. gigantea* was occupied by supporting tissue.

**Figure 7:**
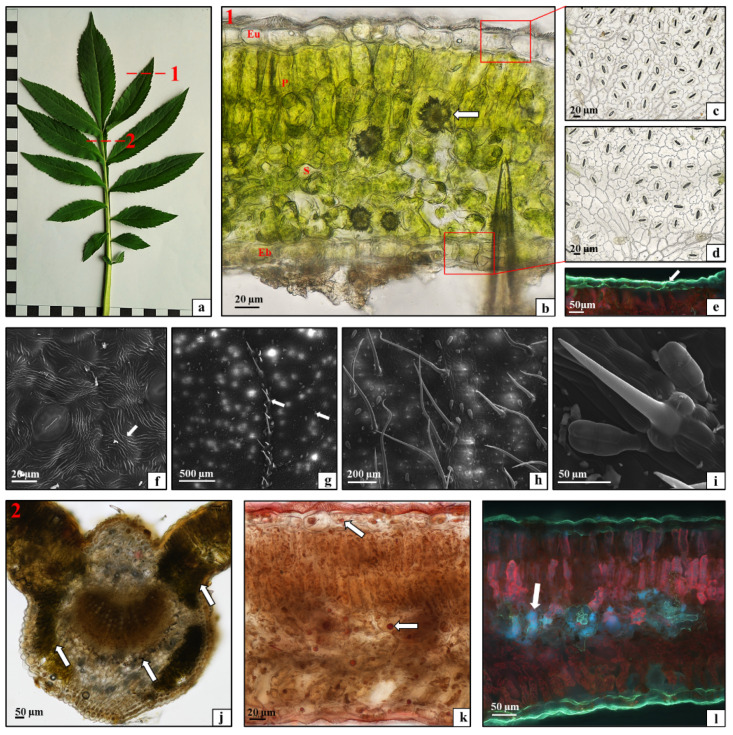
Morphology and anatomy of a *Cephalaria gigantea* leaf: (**a**) macroscopic view, dotted line (1 and 2)—histologically analyzed fragments; (**b**) cross-section through the leaf blade: upper epidermis (Eu), palisade mesophyll (P), spongy mesophyll (S), calcium oxalate crystal (arrow), bottom epidermis (Eb); (**c**) abaxial leaf epidermis; (**d**) adaxial leaf epidermis; (**e**) cuticle layer on the abaxial leaf epidermis—white arrow; (**f**) cuticle ornamentation on the abaxial leaf epidermis (arrow); (**g**) trichomes on the abaxial leaf epidermis (arrows); (**h**,**i**) trichomes on the adaxial leaf epidermis; (**j**) starch grains (arrows) in the midrib and leaf mesophyll; (**k**) oleosomes (arrows) in the leaf blade; (**l**) phenolic compounds (arrow) in the leaf mesophyll ((**b**–**d**,**j**,**k**) light microscope; (**e**,**l**) fluorescence microscope; (**f**–**i**) SEM).

The leaves of *C. gigantea* were analyzed in this study as well. These were pinnate leaves with serrated margins (Figure 7a). The analysis of their histological structure showed that the lamina consisted of a single-layered upper epidermis (Figure 7b—Eu), under which there were two layers of palisade mesophyll (Figure 7b—P). The next layer was the spongy mesophyll (Figure 7b—S) with numerous calcium oxalate crystals (Figure 7b—arrow). The lower epidermis was also composed of one layer of closely adjacent cells (Figure 7b—Eb). The peel preparations of the epidermis from the abaxial and adaxial surface of the lamina showed cells with a parentimatic polyhedral shape, between which there were numerous diacytic Amaryllis-type stomata (Figure 7c,d). This tissue was covered by a cuticle (Figure 7e—white arrows) with characteristic linear ornamentation (Figure 7f—white arrow). The epidermis on both lamina surfaces had two types of trichomes: pointed unicellular mechanical trichomes and multicellular glandular trichomes composed of a single-celled base and a four-celled head (Figure 7g–i). It was observed that the mechanical trichomes on the adaxial surface of the lamina were part of the epidermis covering the leaf veins, and the glandular trichomes were present on the entire lamina surface (Figure 7g). In turn, the epidermis on the entire abaxial surface had both types of trichomes (Figure 7h,i). As shown in the present study, the *C. gigantea* leaves are not isolateral and have palisade mesophyll only on the adaxial surface, but the epidermis cells are covered by a thicker layer of strongly ornamented cuticle than that observed in *C. uralensis*. Thus, the species differ slightly in their leaf anatomy-related strategy of protection against excessive radiation [29,35]. In turn, the presence of trichomes and numerous calcium oxalate crystals was similar in both species. The cytochemical studies of the *C. gigantea* leaf tissues demonstrated the accumulation of small amounts of starch in the midrib, in the parenchyma immediately below the epidermis, around the vascular bundle, and in the mesophyll (Figure 7j—arrows). This indicates that this primary metabolite was present in the same leaf tissues as in *C. uralensis*, but its amounts were substantially smaller. In contrast, a relatively large number of oleosomes containing sudanophilic lipids was observed in the lamina tissues, mainly in the spongy mesophyll and leaf epidermis in *C. gigantea* (Figure 7k—arrows). The presence of the relatively large number of oleosomes storing photosynthetic products and their properties useful for advanced and broad applications [36] indicate that the species has high application potential related to the content of bioactive substances. The analysis of the distribution of phenolic compounds in the leaf tissues showed large amounts of these substances accumulated mainly in the spongy mesophyll of the leaf blade (Figure 7d—arrow).

The analyses of both species provide a conclusion that *C. gigantea* leaves may be a richer source of phenolic compounds, as they have a thicker layer of spongy mesophyll than *C. uralensis* leaves. It should be emphasized that phenolic substances are part of the natural defense mechanism during plant exposure to stress conditions, e.g., oxidative stress, and are involved in protection against various types of intruders. Importantly, with their biological parameters, phenolic compounds are broadly used in pharmacy [37]; therefore, the knowledge of their distribution and correlation with individual plant elements provides valuable information for efficient extraction of these compounds. It should be underlined that there is little information about the distribution of phenolic compounds in the tissues of individual plant organs in the studied species. Therefore, the present study not only complements anatomical/histological plant analyses, but also extends our knowledge of the relationship between the content of bioactive substances and the anatomical structure of the plant.

### 2.3. Phytochemical Analysis

**Figure 8:**
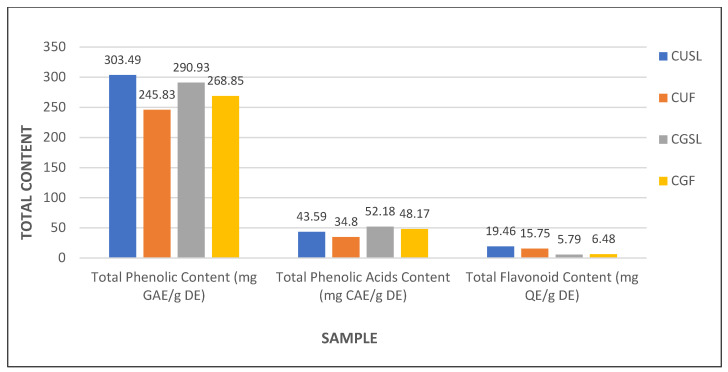
Comparison of the total phenolic content (TPC), total phenolic acid content (TPAC), and total flavonoid content (TFC) in the extracts from *C. uralensis* and *C. gigantea* stems, leaves, and flowers. Values are expressed as mg of gallic acid equivalents (GAE), caffeic acid equivalents (CAE), and quercetin equivalents (QE) per g of dry extract (DE). CUSL—*C. uralensis* stems and leaves, CUF—*C. uralensis* flowers, CGSL—*C. gigantea* stems and leaves, CGF—*C. gigantea* flowers.

To examine the potentially active compounds in the stems, leaves, and flowers of *C. uralensis* and *C. gigantea*, the total content of phenolic compounds, flavonoids, and phenolic acids was determined spectrophotometrically from extracts obtained from particular plant elements. Phenolic compounds, e.g., flavonoids (present as aglycones, glycosides, and methylated derivatives) and phenolic acids, are commonly known as secondary metabolites of plants [37]. Phenolic compounds often occur as water-soluble molecules; therefore, they are mainly stored in plant vacuoles, which are the main reservoirs of biologically active compounds used in medical applications [38]. The total phenolic content (TPC) was examined using the Folin–Ciocalteu reagent and the results were expressed as gallic acid equivalents (GAE) per g of dry extract. The TPC value in the stems and leaves was comparable, i.e., 303.49 ± 0.05 mg GAE/g DE in *C. uralensis* and 290.93 ± 0.03 mg GAE/g D.E in *C. gigantea* (Table 1, Figure 8). In turn, the TPC in aboveground shoots in the other *Cephalaria* species studied was 2.658 ± 0.1 mg GAE/g DE in *C. speciosa* and 3.037 ± 0.156 mg GAE/g DE in *C. tchichatchewii* [39]. However, the method used in the current report is based on the usage of the methanolic solvent, contrary to the method based on hexane used in the previous report [39]. It should be added that, due to the presence of fatty acids and other apolar components in *n*-hexane extracts, the TPC level is usually lower than in methanolic extracts, which contain more phenolic and polar compounds. Thus, our analyses provide a more precise estimation of TPC. Therefore, we can indicate that the *C. uralensis* and *C. gigantea* plants analyzed in this study had substantially higher phenolic content in the stems and leaves. The content of these bioactive substances in the flowers of both studied species was high in both *C. uralensis* (245.83 ± 0.02 mg GAE/g DE) and *C. gigantea* (268.85 ± 0.03 mg GAE/g DE) (Table 1, Figure 8). Importantly, there have been no reports of the TPC content in the flowers in other species from the genus *Cephalaria* to date, indicating that they may be a superb but unexplored source of TPC and emphasizing the role of these plant elements as a new source of bio-compounds in these species.

Phenolic acids constitute a dominant group of phenolic compounds that occur in plants and their high content is desirable from the medical point of view, due to their strong antioxidant activity preventing many serious diseases related to oxidative stress [37,40]. It should be stressed that the total phenolic acid content (TPAC) in individual aboveground organs in plants belonging to *Cephalaria* species has never been studied. In this report, we performed the measurement of TPAC and the content was expressed in mg of caffeic acid (CAE) per g of dried extract. These analyses showed that the TPAC in the stems and leaves was higher in *C. gigantea* (52.18 ± 0.06 mg CAE/g DE) than in the same organs of *C. uralensis* (43.59 ± 0.05 mg CAE/g DE). Similarly, the amounts of total phenolic acids contained in the flowers were higher in *C. gigantea* (48.17 ± 0.02 mg CAE/g DE) than in *C. uralensis* (34.80 ± 0.01 mg CAE/g DE) (Table 1, Figure 8). Interestingly, our analyses indicate that all organs of *C. gigantea* have higher TPAC values than *C. uralensis*.

Other compounds that are important medicinal constituents are flavonoids, occurring mainly as aglycones, glycosides, and methylated derivatives representing a large group of phenolic compounds [37]. With their strong antioxidant activity, these compounds participate in many different reactions, such as the protection of lipids or other important cell molecules against oxidation [41]. Therefore, the total flavonoid content (TFC) in the stems, leaves, and flowers of *C. uralensis* and *C. gigantea* was investigated and expressed as mg quercetin per g of dry extract. The total flavonoid content in the stems and leaves was considerably higher in *C. uralensis* (19.46 ± 0.02 mg QE/g DE) than in *C. gigantea* (5.79 ± 0.03 mg QE/g DE). Literature reports on the content of flavonoids in stems and leaves determined only in *C. gigantea* indicate a relatively low level of these substances (7.8 ± 2.1 mg QE/g DE) in aboveground organs in this species [42]. Thus, it can be assumed that *C. gigantea* leaves and stems are less valuable as a source of flavonoids than the same organs of *C. uralensis*. Notably, the flavonoid content in *C. uralensis* and *C. gigantea* flowers was investigated as well, as no such data have been reported to date. The study showed a high content of these compounds in these organs, and greater amounts of flavonoids were found in the *C. uralensis* flowers (15.75 ± 0.02 mg QE/g DE) than in the *C. gigantea* flowers (6.48 ± 0.01 mg QE/g DE). These results once again showed that the content of flavonoids in the aboveground shoots of *C. uralensis* is clearly higher than that in *C. gigantea*; hence, *C. uralensis* flowers can especially be regarded as a valuable source of flavonoids (Table 1, Figure 8).

To sum up, we undertook the comprehensive study of the two species *C. gigantea* and *C. uralensis*, which represent a highly interesting and concurrently unexplored area with respect to medicinal applications. The species analyzed in this study were used to determine the content of total phenolic compounds, total phenolic acids, and total flavonoids, especially considering their distribution in individual organs. It should be emphasized that the levels of the analyzed substances were high in comparison with many other medicinal plants, making *C. gigantea* and *C. uralensis* plants a very promising source of biologically active compounds. As indicated in the present study, *C. uralensis* seems to be a more attractive species than *C. gigantea*, primarily due to its high total phenolic content and total flavonoid content, which makes it an interesting object for further searches for new natural substances with medical applications. It should be stressed that polyphenols are an important part of the human diet, with a critical impact on cell metabolism [43]. These so-called biologically active substances contained in plants (especially in a high proportion in *C. gigantea* and in *C. uralensis*) protect against oxidative stress, which has a deleterious influence on the major cellular compounds such as lipids, proteins, and other molecules in human cells that may undergo unspecific oxidation. Thus, these species represent a highly attractive unexplored territory in this regard [44]. Additionally, it has frequently been reported that these phytochemicals display antibacterial [45], anti-inflammatory [46], and immunostimulant activity [47], thus playing an important role in the treatment of various infections and in the determination of the human microbiome. Such substances as flavonoids and phenolic acids have numerous applications in the treatment of several human diseases, e.g., neoplasms [48], neurodegenerative disorders [49], cardiovascular diseases [50], and arthritis [51]. The compounds were found to be very efficient to combat acne and other skin problems caused by microbial infections, i.e., diseases that are very difficult to treat due to the low repertoire of adequate antibiotics [52]. Therefore, the present characterization of the *C. uralensis* and *C. gigantea* species, which have never been described in terms of their anatomy, histology, and cytology in relation to the content of bioactive substances in individual organs, extends the knowledge of the medicinal values of these plants, and fits in with the trend in the search of new species with medicinal applications.

## 3. Materials and Methods

### 3.1. Chemicals and Reagents

The Folin–Ciocalteu reagent, ethylenediaminetetraacetic acid, and disodium dihydrate (Na_2_EDTA×2H_2_O) were obtained from Sigma-Aldrich (Steinheim, Germany). Reference substances were purchased from ChromaDex (Irvine, CA, USA). All other chemicals were of analytical grade and were purchased from Polish Chemical Reagent Company (POCH, Gliwice, Poland).

### 3.2. Plant Material

The research plants *Cephalaria uralensis* (Murray) Roem. & Schult. and *C. gigantea* (Ledeb.) Bobrov were collected from the Botanical Garden of Maria Curie-Skłodowska University in Lublin. The Botanical Garden is situated in the NW part of Lublin (latitude 51°16′ N and longitude 22°30′ E). Brown soils prevail in the area of the Botanical Garden (Index Seminum 2014 Hortus Botanicus Universitatis Marie Curie-Skłodowska). The cultivation was carried out in a univariate randomized block design with 4 replications. Observations of the developmental phases and morphological traits of *C. uralensis* and *C. gigantea* as well as plant collection were carried out in 2019 and 2020 (from April to September). No herbicides, fungicides, or any chemicals were used during cultivation, and manual weeding methods were used throughout the growing period. Macroscopic images of the plants were taken with a Nikon D300 photographic camera equipped with a 60 mm AF MICRO NIKKOR lens.

### 3.3. Light Microscopy (LM)

Aboveground shoots of *C. uralensis* and *C. gigantea* were collected for histological and cytochemical analyses. The material was sampled randomly from 20 plants. The analyses were performed using fresh fragments of leaves and stems. They were used for making manual cross-sections and peel preparations of the leaf epidermis. Starch was visualized by staining the sections placed on slides with Lugol’s solution (KJ was dissolved in distilled water, final concentration 2%) for 15 min, then rinsed with distilled water. The starch grains stained black. Sudanophilic fats were detected and localized with the use of a Sudan IV solution mixed with glycerol in 1:1 ratio; subsequently, the tissue sections were covered with a drop of the solution, heated over a flame for 5 min, and rinsed with distilled water. Fat droplets stained orange [53]. The observations of fresh unstained and stained slides were carried out using a Nikon eclipseNi light microscope. Photographic documentation was carried out with a digital camera and NIS-Elements BP software.

### 3.4. Fluorescence Microscope (FM)

Hand-made cross sections were prepared to visualize the cuticle on the epidermis of *C. uralensis* and *C. gigantea* leaves and stems. They were placed in a 0.02% auramine 0 solution in TRIS buffer for several minutes and then rinsed with distilled water [54]. To visualize phenolic compounds, fresh, hand-cut preparations were placed in distilled water and the blue autofluorescence of these compounds was observed [55]. The slides were analyzed under a fluorescence microscope Nikon eclipseNi-u at an excitation wavelength of 330–380 nm and an emission wavelength over 480 nm (UV). Photographic documentation was carried out with a digital camera and NIS-Elements BP software.

### 3.5. Scanning Electron Microscope (SEM)

Fragments of leaves of both species were collected for the analysis of the organ surface (from the top and bottom of the leaf lamina). Freshly collected samples were fixed overnight in 2.5% glutaraldehyde in 0.2 M sodium phosphate buffer (pH 7.4), washed with distilled water, and dehydrated in increasing concentrations of ethanol [56]. The dehydrated samples were then dried in a Critical Point Dryer (Denton Vacuum, Moorestown, NJ, USA) using liquid CO_2_. The dried samples were mounted on aluminum stubs and sputter-coated with gold (Hummer 6.2 Sputter Coater, Anatech USA, Union City, CA, USA). The samples were analyzed under a scanning microscope (LEO1430VP) with an accelerating voltage of 15 kV equipped with a Bruker Ouantax 200XFlash EDX Spectrometer System attached to a Zeiss EVO 50 Variable Pressure SEM at 15 kV, using INCA-Mapping software (Billerica, MA, USA).

### 3.6. Preparation of the Extracts

The aboveground shoots of the plants were collected for phytochemical analyses in July–August. The plant material was divided into two groups: one group consisted of stems and leaves and the other group comprised flowers at full anthesis. Next, the plant materials were shade-dried at 25 °C (±0.5 °C) until constant weight [57]. A measure of 50 g of powdered stems and leaves and 10 g of flowers were weighted and extracted with sonication with a mixture of methanol-acetone-water (3:1:1, *v/v/v*; 3 × 300 mL and 3 × 100 mL, respectively). After sonication at controlled temperature (40 ± 2 °C) for 30 min, the combined extracts were filtered, concentrated under reduced pressure, and lyophilized in a vacuum concentrator after freezing (Free Zone 1 apparatus; Labconco, Kansas City, KS, USA) to obtain dried residues. Dry extracts were weighted and stored in a freezer at −20 °C. The obtained yields were: *C. gigantea* stems and leaves—10.3 g; *C. gigantea* flowers—2.9 g; *C. uralensis* stems and leaves—10.1 g; and *C. uralensis* flowers—2.3 g. For phytochemical analysis, 100 mg of crude extracts were dissolved in 10 mL methanol and filtered to give stock solutions (10 mg/mL).

### 3.7. Total Phenolic, Flavonoid, and Phenolic Acid Content

Total flavonoid content (TFC) and total phenolic content (TPC) were established using the colorimetric assays as described previously [58]. The absorbance was measured at 430 and 680 nm, respectively, using a Pro 200F Elisa Reader (Tecan Group Ltd., Männedorf, Switzerland). The total phenolic concentration was estimated from the calibration curve (R^2^ = 0.9811) using gallic acid (0.002–0.1 mg/mL) as a standard. The results were expressed as mg of gallic acid equivalents (GAE) per 1 g of dry extract (DE). The total flavonoid content was estimated from the calibration curve (R^2^ = 0.9628) using quercetin (0.03125–2.0 mg/mL) as a standard. The results were expressed as mg of quercetin equivalents (QE) per 1 g of DE. The total phenolic acid content (TPAC) was assayed using Arnov’s reagent as described in Polish Pharmacopoeia IX (an official translation of PhEur 7.0) [57]. The absorbance was measured at 490 nm. TPAC was estimated from the calibration curve (R^2^ = 0.9925) using caffeic acid at a concentration 0.03125–2.0 mg/mL as a standard. The results were expressed as mg of caffeic acid equivalents (CAE) per 1 g of DE.

## 4. Conclusions

As a continuation of the search for new bioactive compound-rich plant species with medicinal applications, the present study focused on the biotechnological aspect of pharmacological use of two plants: *C. uralensis* and *C. gigantea*. The research consisted of a comprehensive analytical approach, including analyses of phenological and morphological traits of the aboveground shoots of these species (leaves, stems, flowers). The analyses did not show major differences between the analyzed *C. uralensis* and *C. gigantea* species and plants growing in different climatic conditions, indicating that the features of these plants are not strictly dependent on climate conditions and can be cultivated in a variety of environmental conditions. Subsequently, as part of the detailed analysis of the plants, histological studies of the aboveground organs were carried out, which showed that the thickness of the supporting tissue layer in *C. uralensis* was relatively small compared to *C. gigantea*, which was undoubtedly associated with the considerable length of the aboveground shoots of the latter species. The cytochemical analyses showed large amounts of starch in both the leaf mesophyll and stem parenchyma in *C. uralensis* and *C. gigantea*, but its amount was substantially lower in *C. gigantea* tissues. Large amounts of sudanophilic lipids were detected only in the spongy parenchyma and leaf epidermis of *C. gigantea*. Subsequently, numerous secondary metabolites were analyzed to determine their distribution in the plant organs. The analyses of phenolic compounds in both species revealed that the spongy parenchyma in the leaf and the primary cortex parenchyma in the stem were the main reservoirs of these secondary metabolites. However, in *C. uralensis* these tissues occupied a bigger area than in *C. gigantea*, which suggests that the *C. uralensis* stem is a better source of these compounds. The quantitative phytochemical analyses of the content of total phenolic, total phenolic acids, and total flavonoids in the aboveground organs of both species showed that the content of flavonoids in *C. uralensis* aboveground shoots was higher than in *C. gigantea*. Interestingly, it was shown that *C. uralensis* flowers were characterized by high content of flavonoids; hence, they can be an excellent source of biologically active substances. The investigations of *C. uralensis* and *C. gigantea* presented in this paper provided a comprehensive assessment of these two species in terms of the effective use of individual parts of these plants for the extraction of biologically active substances, indicating their enormous but unexplored pharmaceutical potential.

## Figures and Tables

**Table 1:** Total phenolic content (TPC), total phenolic acid content (TPAC), and total flavonoid content (TFC) in the stems, leaves, and flowers of *C. uralensis* and *C. gigantea*. The results are expressed as mg of gallic acid equivalents (GAE), caffeic acid equivalents (CAE), and quercetin equivalents (QE) per g of dry extract (DE). CUSL—*C. uralensis* stems and leaves, CUF—*C. uralensis* flowers, CGSL—*C. gigantea* stems and leaves, CGF—*C. gigantea* flowers.

Sample	Total Phenolic Content (mg GAE/g DE)	Total Phenolic Acids Content (mg CAE/g DE)	Total Flavonoid Content (mg QE/g DE)
CUL	303.49 ± 0.05	43.59 ± 0.05	19.46 ± 0.02
CUF	245.83 ± 0.02	34.80 ± 0.01	15.75 ± 0.02
CGL	290.93 ± 0.03	52.18 ± 0.06	5.79 ± 0.03
CGF	268.85 ± 0.03	48.17 ± 0.02	6.48 ± 0.01

Values are presented as mean ± SD, *n* = 5.
